# Parity and Pancreatic Cancer Risk: A Dose-Response Meta-Analysis of Epidemiologic Studies

**DOI:** 10.1371/journal.pone.0092738

**Published:** 2014-03-21

**Authors:** Hong-Bo Guan, Lang Wu, Qi-Jun Wu, Jingjing Zhu, Tingting Gong

**Affiliations:** 1 Department of Obstetrics and Gynecology, Shengjing Hospital, China Medical University, Shenyang, China; 2 Center for Clinical and Translational Science, Mayo Clinic, Rochester, Minnesota, United States of America; 3 Department of Epidemiology, Shanghai Cancer Institute, Renji Hospital, Shanghai Jiaotong University School of Medicine, Shanghai, China; 4 State Key Laboratory of Oncogene and Related Genes, Shanghai Cancer Institute, Renji Hospital, Shanghai Jiaotong University School of Medicine, Shanghai, China; 5 Department of Educational Psychology, University of Minnesota, Minneapolis, Minnesota, United States of America; Centro Nacional de Investigaciones Oncológicas (CNIO), Spain

## Abstract

**Background:**

Previous epidemiologic studies have reported inconsistent results between parity and pancreatic cancer (PC) risk. To our knowledge, a comprehensive and quantitative assessment of this association has not been conducted.

**Methods:**

Relevant published studies of parity and PC were identified using MEDLINE (PubMed) and Web of Science databases until November 2013. Two authors (H-BG and LW) independently assessed eligibility and extracted data. Eleven prospective and 11 case-control studies reported relative risk (RR) estimates and 95% confidence intervals (CIs) of PC associated with parity. Fixed- and random-effects models were used to estimate the summary RR depending on the heterogeneity of effects.

**Results:**

The summary RR for PC comparing the highest versus lowest parity was 0.86 (95% CI: 0.73–1.02; *Q* = 50.49, *P*<0.001, *I* ^2^ = 58.4%). Significant inverse associations were also observed in the studies that adjusted for cigarette smoking (RR = 0.81; 95% CI: 0.68–0.98), Type 2 diabetes mellitus (RR = 0.83; 95% CI: 0.75–0.93), and those that included all confounders or important risk factors (RR = 0.85; 95% CI: 0.76–0.96). Additionally, in the dose-response analysis, the summary RR for per one live birth was 0.97 (95% CI: 0.94–1.01; *Q* = 62.83, *P*<0.001, *I* ^2^ = 69.8%), which also indicated a borderline statistically significant inverse effect of parity on PC risk. No evidence of publication bias and significant heterogeneity between subgroups were detected by meta-regression analyses.

**Conclusion:**

In summary, these findings suggest that higher parity is associated with a decreased risk of PC. Future large consortia or pooled studies are warranted to fully adjust for potential confounders to confirm this association.

## Introduction

Pancreatic cancer (PC) is the fourth leading cause of cancer death for both sexes in the United States [1], although the incidence rate is 30–50% higher in men than women [2]. Based on current knowledge, the incidence rate difference between the sexes cannot be completely attributed to established risk factors including cigarette smoking, overweight and obesity, history of diabetes mellitus (DM), history of pancreatitis, and non-O blood group [2]. Given the evidence from *in vitro* and *in vivo* studies [3]–[4], it has been hypothesized that hormone-related or reproductive factors related to estrogen exposure may play a role in the etiology of PC.

Among the reproductive factors that have been investigated, parity (the number of livebirths in a woman's lifetime), is less likely to be prone to recall bias and misclassification [5], but the results of epidemiologic studies have been conflicting which might be attributed to limited statistical power or inadequate adjustment for confounders [6]–[32]. Though a previous review has focused on this topic [33], to our knowledge, a comprehensive and quantitative assessment of the association between parity and PC risk has not been conducted. Therefore, we carried out a dose-response meta-analysis on epidemiologic studies published up to November 2013 to quantify the association between parity and PC.

## Materials and Methods

### Literature search

We carried out a comprehensive literature search using MEDLINE (PubMed) and Web of Science from database initiation until November 12, 2013. The search was limited to published studies of humans by using the following search key words and Medical Subject Headings terms: (parity OR pregnancy OR livebirth OR reproductive OR reproduction OR reproductive factors) AND (pancreas OR pancreatic) AND (cancer OR neoplasm OR carcinoma OR tumor). We also reviewed the references of all included studies for additional publications. We followed PRISMA (Preferred Reporting Items for Systematic Reviews and Meta-Analyses) for conducting and reporting meta-analyses [34]–[36].

### Study selection criteria

**Figure 1:**
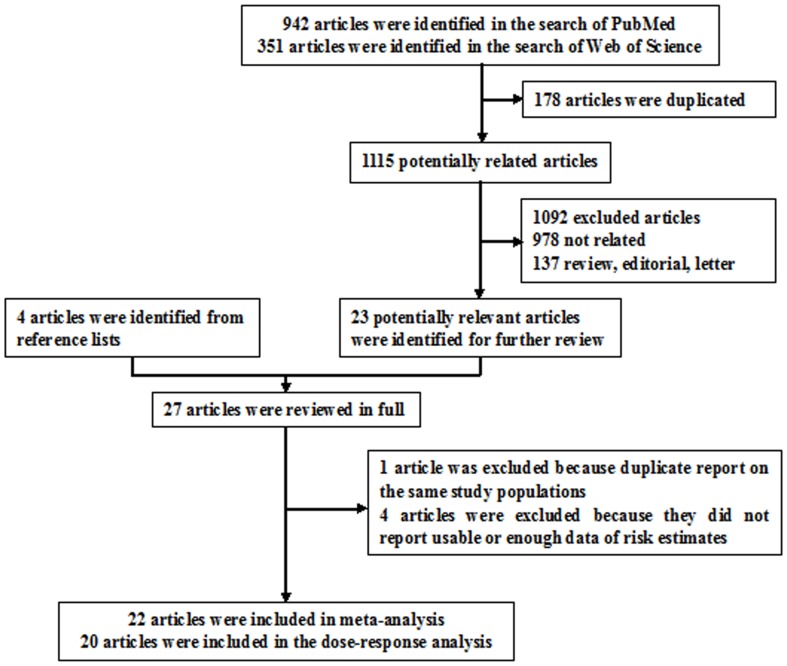
Selection of studies for inclusion in meta-analysis.

Published studies were included if they 1) used a case-control or prospective study design; 2) evaluated the association between parity and PC risk; and 3) presented hazard ratio (HR), odds ratio (OR), or relative risk (RR) estimates with 95% confidence intervals (CI), standard errors (SE) or data necessary to calculate these. When multiple publications from the same study were available, we used the publication with the largest number of cases and most applicable information. After excluding 1045 and 47 articles based on screening of titles or abstracts, respectively, we identified 27 potentially relevant articles for further full text review (Figure 1) [6]–[32]. One article was excluded because of duplicate reports from the same study population [29], 4 articles were excluded because they did not report usable or enough data of risk estimate [26], [28], [31]–[32]. Since less than 5% of the PC patients survive more than 5 years after diagnosis, thus we did not excluded the studies [9], [12], [16]–[17] reported the risk estimates between parity and PC mortality.

### Data abstraction

For each eligible study, two investigators (H-BG and LW) independently performed the eligibility evaluation and data abstraction. The disagreements were discussed and resolved by consensus or by involving a third reviewer (Q-JW) for adjudication. Data abstracted from each study were: author list, year of publication, study region and design, study sample size (number of cases and controls or cohort size), range of follow-up for cohort studies, exposure and outcome assessment including parity categories, study-specific adjusted estimates with their 95% CIs for the highest versus lowest parity, and factors matched by or adjusted for in the design or data analysis. If multiple estimates of the association were available, we abstracted the estimate that adjusted for the most covariates.

### Statistical analysis

The study-specific adjusted RRs were used as the measure of association across studies. Because the absolute risk of PC is low, we assumed that estimates of ORs from case-control studies and risk, rate or hazard ratios from prospective studies were all valid estimates of the RR and we therefore report all results as the RR for simplicity. For studies that did not use the category with lowest parity as the reference [11], [19], [23], we used the effective count method proposed by Hamling et al [37] to recalculate the RRs.

For the dose-response analysis which calculated parity as a continuous variable, we used the method proposed by Greenland et al. [38] to compute study-specific slopes (linear trends) and 95% CIs from the natural logs of the RRs and CIs across categories of parity. This method requires that the distribution of cases, person-years of non-cases and the RRs with the variance estimates for at least three quantitative exposure categories are known. For studies that reported the parity number by ranges we estimated the midpoint in each category by calculating the average of the lower and upper bound. When the highest category did not have an upper bound we assumed that the width of the open ended interval was the same as that of the adjacent interval. When the lowest category did not have a lower bound we set the lower bound to zero. The dose-response results in the forest plots are presented based on increments of 1 live birth for parity. We examined a potential nonlinear dose-response relationship between parity and PC using fractional polynomial models [39]. We determined the best-fitting second-order fractional polynomial regression model as the one with the lowest deviance. A likelihood ratio test was used to assess the difference between the nonlinear and linear models to test for nonlinearity [40].

We evaluated heterogeneity of RRs across studies by using the Cochrane *Q* statistic and the *I* ^2^ statistic [41]. For the *Q* statistic, a *P*-value less than 0.1 was considered to be representative of statistically significant heterogeneity. *I* ^2^ represents the proportion of total variation contributed by between-study variation [41]. The summary estimate was based on the fixed-effects model [41] when no heterogeneity was detected or the random-effects model [42] when substantial heterogeneity was detected. In both methods, the study weight was the inverse of the variance of log RR, which was estimated from the 95% CI from each study. Subgroup analyses were carried out based on study design (cohort vs. case-control studies), number of cases (≥200 (median) vs. <200), type of controls for the case-control studies (population-based vs. hospital-based controls), geographic location (North America, Europe, or Asia). We also stratified the meta-analysis by potentially important confounders (i.e., body mass index (BMI), cigarette smoking, and Type 2 DM). Heterogeneity between subgroups was evaluated by meta-regression. A *P*-value less than 0.05 for meta-regression was considered representative of significant statistical difference between subgroups. Finally, we carried out sensitivity analyses excluding one study at a time to explore whether the results were strongly influenced by a specific study.

Publication bias was evaluated via Egger's linear regression [43], Begg's rank correlation methods [44] and funnel plots. A *P*-value less than 0.05 for Egger's or Begg's tests was considered representative of significant statistical publication bias. Statistical analyses were performed with Stata (version 11.2; StataCorp, College Station, TX). *P*-values were two sided with a significance level of 0.05.

## Results

### Study characteristics

Characteristics of the 22 included articles [6]–[25], [27], [30] are shown in Table S1. The included articles, which represent 8,247 cases and 3,498,673 non-cases, were published between 1992 and 2013 and consist of 11 prospective studies (9 cohort studies [7], [9], [12]–[13], [15]–[18], [20] and two nested case-control studies [6], [23]) and 11 case-control studies [8], [10]–[11], [14], [19], [21]–[22], [24]–[25], [27], [30]. Of the 11 prospective studies, four were conducted in the United States [7], [15], [17], [20], and one each in Taiwan [9], United Kingdom [12], Norway [13], Japan [16], Canada [18], Sweden [23], and an international group which covered multiple countries [6]. Cohort sizes ranged from 37,459 [15] to 1,292,462 [9], and the number of PC cases varied from 154 [16] to 1,959 [17]. The highest parity in the prospective studies varied from 3 [9] to over 7 [17].

Of the 11 case-control studies, three were conducted in the United States [10], [19], [27], two each in Italy [8], [25] and Canada [21]–[22], and one each in Egypt [14], China [24], and a study covering multiple countries [11]. The number of cases enrolled in these studies ranged from 56 [45] to 608 [46], and the number of control subjects varied from 52 [21] to 367 [11]. Control subjects were drawn from the general population in 7 studies [11], [19], [21]–[22], [24], [27], [30], hospitals in 4 studies [8], [10], [14], [25], The highest parity in the case-control studies varied from 3 [11] to over 7 [14].

### High vs. low parity

**Figure 2:**
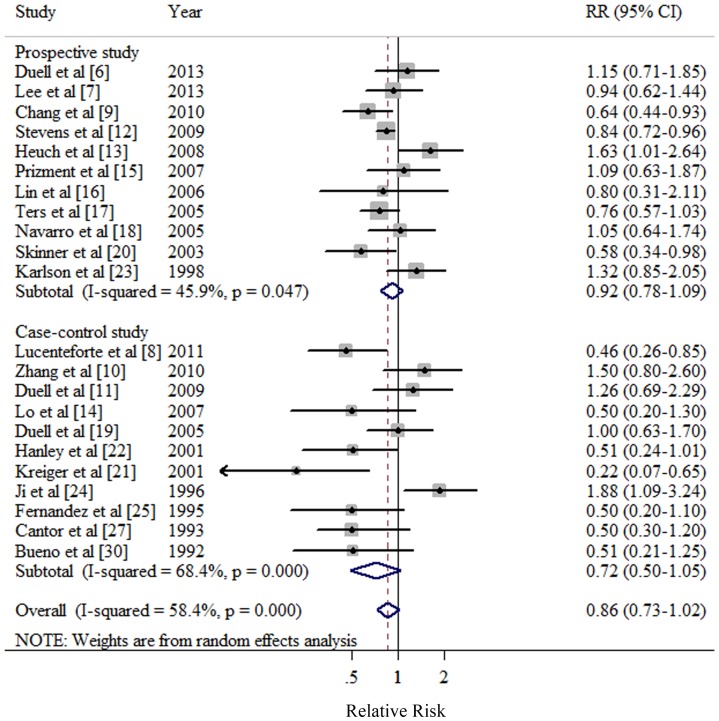
Forest plot (random effects model) of parity (highest versus lowest) and pancreatic cancer risk by study design. Squares indicate study-specific relative risks (size of the square reflects the study-specific statistical weight); horizontal lines indicate 95% CIs; diamond indicates the summary relative risk estimate with its 95% CI. CI: confidence interval; RR: relative risk.

Eleven prospective [6]–[7], [9], [12]–[13], [15]–[18], [20], [23] and 11 case-control studies [8], [10]–[11], [14], [19], [21]–[22], [24]–[25], [27], [30] investigated the association between parity and PC risk. The summary RR of PC for the highest vs. lowest categories of parity was 0.86 (95% CI: 0.73–1.02), with significant heterogeneity (*Q* = 50.49, *P*<0.001, *I* ^2^  = 58.4%) (Table 1 and Figure 2). There was no indication of publication bias with Egger's test (*P* for bias  = 0.652) or with Begg's test (*P* for bias  = 0.324) and no asymmetry was seen in the funnel plots when inspected visually. In a sensitivity analysis, we sequentially removed one study at a time and re-analyzed the data. The 22 study-specific RRs of parity ranged from a low of 0.84 (95% CI: 0.71–0.98, *Q* = 42.53, *P* = 0.002, *I* ^2^  = 53.0%) after omission of the study by Ji et al [24] to a high of 0.89 (95% CI: 0.76–1.05, *Q* = 45.98, *P* = 0.001, *I* ^2^  = 56.5%) after omission of the study by Lucenteforte et al. [8].

**Table 1:** Summary risk estimates of the association between parity and pancreatic cancer risk.

	Highest versus lowest						Dose-response analysis (per 1 live birth)					
	No. of	Summary RR	*Q*	*I* ^2^ Value	*P*_h_ *	*P*_h_ **	No. of	Summary RR	*Q*	*I* ^2^ Value	*P*_h_ *	*P*_h_ **
	studies	(95% CIs)	Statistic	(%)			studies	(95% CIs)	Statistic	(%)		
**Overall**	22	0.86 (0.73–1.02)	50.49	58.4	<0.001	—	20	0.97 (0.94–1.01)	62.83	69.8	<0.001	
**Subgroup analyses**												
Study Design						0.62						0.14
Prospective studies	11	0.92 (0.78–1.09)	18.5	45.9	0.047		9	0.99 (0.95–1.03)	31.29	74.4	<0.001	
Case-control studies	11	0.72 (0.50–1.05)	31.64	68.4	<0.001		11	0.95 (0.90–1.01)	25.51	60.8	0.004	
Number of cases						0.08						0.18
<200	10	0.71 (0.49–1.04)	24.19	62.8	0.004		10	0.95 (0.90–1.01)	23.11	61.1	0.006	
≥200	12	0.92 (0.77–1.10)	26.15	57.9	0.0006		10	0.98 (0.95–1.02)	35.45	74.6	<0.001	
Type of Control Subjects						0.25						0.38
Population based	7	0.75 (0.46–1.22)	21.24	71.8	0.002		7	0.94 (0.85–1.04)	20.59	70.9	0.002	
Hospital based	4	0.67 (0.35–1.27)	9.44	68.2	0.024		4	0.96 (0.92–1.02)	4.80	37.6	0.187	
Geographic location						0.27						0.33
North America	10	0.81 (0.63–1.02)	17.93	49.8	0.036		10	0.97 (0.94–1.01)	17.3	48.0	0.044	
Europe	7	0.89 (0.65–1.22)	18.91	68.3	0.004		5	0.97 (0.90–1.04)	15.27	73.8	0.004	
Asia	3	0.99 (0.46–2.13)	10.27	80.5	0.006		3	0.99 (0.86–1.14)	10.27	80.5	0.006	
**Adjustment for confounders or important risk factors**												
BMI						0.19						0.16
Yes	10	0.89 (0.71–1.12)	24.2	62.8	0.004		9	0.99 (0.94–1.03)	21.58	62.9	0.006	
No	12	0.82 (0.63–1.08)	26.29	58.2	0.006		11	0.96 (0.91–1.01)	33.19	69.9	<0.001	
Cigarette smoking						0.70						0.90
Yes	18	0.81 (0.68–0.98)	36.71	53.7	0.004		17	0.97 (0.94–1.00)	30.30	47.2	0.016	
No	4	1.09 (0.71–1.68)	10.98	72.7	0.012		3	0.99 (0.90–1.08)	11.23	82.2	0.004	
DM						0.32						0.29
Yes	8	0.83 (0.75–0.93)	11.93	41.3	0.103		7	0.98 (0.96–1.00)	7.21	16.7	0.302	
No	14	0.86 (0.65–1.13)	37.20	65.1	<0.001		13	0.97 (0.92–1.02)	43.14	72.2	<0.001	
BMI, Cigarette smoking, and Type 2 DM				0.16						0.16		
Yes	6	0.85 (0.76–0.96)	7.89	36.6	0.162		5	0.98 (0.96–1.00)	4.85	17.5	0.303	
No	18	0.82 (0.63–1.06)	42.39	64.6	<0.001		15	0.96 (0.92–1.01)	49.65	71.8	<0.001	

RR: relative risk; CI: confidence interval; BMI: body mass index; DM: diabetes mellitus.

* P value for heterogeneity within each subgroup.

^**^ P value for heterogeneity between subgroups with meta-regression analysis.

### Dose-response analysis

Nine prospective [6]–[7], [9], [15]–[18], [20], [23] and 11 case-control studies [8], [10]–[11], [14], [19], [21]–[22], [24]–[25], [27], [30] were included in the dose-response analysis. The summary RR per live birth was 0.97 (95% CI: 0.94–1.01), with significant of heterogeneity (*Q* = 62.83, *P*<0.001, *I* ^2^  = 69.8%) (Table 1). In a sensitivity analysis excluding one study at a time, the summary RR for PC ranged from 0.97 (95% CI: 0.94–0.99, *Q* = 58.98, *P*<0.001, *I* ^2^  = 69.5%) when the study by Ji et al [24] was excluded to 0.98 (95% CI: 0.95–1.01, *Q* = 56.29, *P*<0.001, *I* ^2^  = 68.0%) when the study by Chang et al [9] was excluded. Additionally, the effect of excluding the two studies not included in the dose-response analysis on the summary RR for high vs. low parity was explored. The summary RR was 0.83 (95% CI: 0.68–1.00, *Q* = 43.60, *P* = 0.001, *I* ^2^  = 56.4%) which was similar to the original analysis including all studies. There was no evidence of a nonlinear association between parity and PC risk, *P* for nonlinearity  = 0.2409. Furthermore, when we removed three studies [9], [16]–[17] which reported the risk estimates from PC mortality, the results (RR, 0.98; 95% CI: 0.94–1.02, *Q* = 49.60, *P*<0.001, *I* ^2^  = 67.7%) were similar.

### Subgroup analyses

In subgroup analyses of highest versus lowest categories of parity and PC risk, almost all strata showed inverse associations, although not all of them showed statistical significance. There was no evidence of significant heterogeneity between subgroups with meta-regression analyses (Table 1). In the analyses stratified by whether the study included adjustment for specific potential confounders or important risk factors, significant inverse associations were observed in studies which adjusted for cigarette smoking, Type 2 DM, or all of the potential confounders (Table 1). In addition, little heterogeneity was observed in the studies which adjusted for these aforementioned risk factors. Similar patterns were also observed in the dose-response analyses, but the results showed borderline statistical significance (Table 1). Since mortality rate of PC could be confounded by survival related factors, we further excluded the mortality estimates by the studies reported the risk estimates with PC mortality [9], [12], [16]–[17], but the estimate (RR  = 0.89) were similar to the original analysis including all studies.

## Discussion

To our knowledge, this is the first meta-analysis which provide comprehensive and quantitative evidence of the association between parity and PC risk. In this study, high parity was associated with a borderline statistically significant decreased risk of PC although the decrease in risk was small for each additional live birth. In addition, consistent inverse associations were observed within the analyses stratified by whether the studies included adjustment for cigarette smoking, Type 2 DM, or all of the confounders or important risk factors (Table 1).

Although the exact biological mechanisms by which increased parity may decrease the risk of PC are not well established, a hypothesis that endogenous estrogens may be protective against PC has been proposed based on in-vitro and in-vivo studies [3], [47]. Pregnancy elevates serum estrogen levels approximately 100-fold [48]. Women with high parity are likely to have had longer periods of exposure to high levels of circulating estrogens which has been shown to inhibit the growth of preneoplastic pancreatic lesions or transplanted pancreatic carcinoma in rodent models [49]–[50]. In addition, sex-steroid biosynthetic enzymes (e.g., pancreatic homogenates and aromatase) and steroid hormone receptors have been detected in both normal and neoplastic human pancreatic tissue [51]–[54]. On the other hand, circulating insulin like growth factors (IGFs), which have been reported to increase PC risk through promoting cellular proliferation and inhibiting apoptosis [55], have been observed to be significantly lower in women with 4 births or more when compared with nulliparous women [56].

Although the results of meta-regression found no evidence of significant heterogeneity between subgroups by study design, the strength of the association estimate for prospective studies was weaker than that from case-control studies although both of them showed inverse associations with PC risk (Table 1). Given the extremely short median survival time of PC cases [57], possible survival bias in case-control studies could be of concern. Furthermore, though parity is less prone to recall bias and misclassification [5], the prospective studies enabled researchers to capture exposure information without the potential biases introduced by using proxy-interviews. For example, Duell et al [11] reported a positive association with PC although the confidence interval included 1. In addition, because deaths from PC may be regarded as a reasonable indicator of the incidence of PC considering about the short survival time and rapidly fatal characteristic of this cancer [9], [57], this meta-analysis included four prospective studies [9], [12], [16]–[17] which reported the risk estimates for PC mortality. Our results were similar when we excluded these four studies in a sensitivity analysis (data not shown).

When we pooled the results stratified by whether the studies included adjustment for confounders, statistically significant inverse associations were detected in studies which adjusted for cigarette smoking, type 2 DM, and those that included all potential confounders. Several established risk factors for PC, including obesity [58]–[59], cigarette smoking [13], [23], and type 2 DM [60]–[61] are associated with parity and therefore might confound the association between parity and PC risk. Although meta-regression found no significant difference between the studies that did or did not adjust for specific confounders, the results and heterogeneity were slightly different. Compared to the high heterogeneity (*Q* = 65.1, P<0.001) which was observed among the studies did not adjust for type 2 DM, the summary results in these studies adjusted for aforementioned confounders had low heterogeneity. Similar pattern was also observed in the studies whether adjusted for the three confounders (BMI, cigarette smoking, and type 2 DM) (Table 1). However, considering that only a third of studies adjusted for type 2 DM and a quarter adjusted for the three confounders, future studies need to carefully adjust for these potential confounders or report analyses stratified by these risk factors to better be able to rule out residual confounding.

This study had several strengths. To the best of our knowledge, this is the first comprehensive and quantitative assessment of parity and PC. Particular strengths of the current meta-analysis are that it including a total of 8,247 cases and 3,498,673 non-cases which should have provided sufficient statistical power to detect this putative association. A further strength is that we carried out a number of subgroup and sensitivity analyses to explore the potential sources of heterogeneity.

Potential limitations of this meta-analysis must be taken into consideration. First, as a meta-analysis of epidemiological studies, this study contains the limitations inherent to combining results from studies with heterogeneous study designs. Cohort studies are less susceptible to bias (e.g., recall and selection bias) than case-control studies because, due to the prospective design, information on exposures is collected before the diagnosis of the disease. Additionally, given the possible differences in detailed confounder adjustment information over time, we also carried out the stratified analysis by published year, but the results were unchanged (data not shown). Second, since we did not have access to the primary data of the studies included in this meta-analysis, we could not perform additional adjustments for potentially important covariates or accurately assign an exposure value to open-ended parity categories [62]. However, this is a common limitation in studies of dose-response relationships based on aggregate data. In addition, since the quality scoring in a meta-analysis of observational studies is controversial, lacks demonstrated validity, and results may not be associated with quality [63], we did not use the Newcastle-Ottawa Scale [64]–[65] to assess the methodological quality of included studies. Instead, we carried out numerous subgroup and sensitivity analyses. Third, significant heterogeneity and possible publication bias must be considered. There was significant heterogeneity for all studies combined in the analysis of high vs. low parity, as well as in the dose-response analysis, which could be explained by many factors, mainly the differences in confounder adjustment (Table 1). In addition, publication bias can be a problem in meta-analyses of published studies; however, we found no statistical evidence of publication bias in this meta-analysis by Egger's linear regression and Begg's rank correlation methods, and there did not appear to be asymmetry in the funnel plots when inspected visually.

In conclusion, this comprehensive meta-analysis provides evidence that increased parity is associated with a slightly decreased risk of PC. Although this relative risk is moderate in size, given the limited established risk factors and low survival rate, further large consortia or pooled studies, are warranted to fully adjust for the potential confounders and focus on the sex-steroid related factors which might play a role in the development of PC malignancies.

## Supporting Information

Table S1 **Characteristics of studies of parity and pancreatic cancer risk.**
(DOC)

Checklist S1 **PRISMA 2009 Checklist.**
(DOC)
