# Supplementary material for: Parity and Pancreatic Cancer Risk: A Dose-Response Meta-Analysis of Epidemiologic Studies
Source: PLoS One. 2014 Mar 21;9(3):e92738. doi: 10.1371/journal.pone.0092738 (PMC3962437; doi:10.1371/journal.pone.0092738)
Supplement: Table S1 — Characteristics of studies of parity and pancreatic cancer risk. (DOC) [file pone.0092738.s001.doc]

**Table S1 Characteristics of studies of parity and pancreatic cancer** risk

| **First author, publication year (reference), Country,** **Study design** | **Cases/subject (age), duration of follow up** | **Parity categories (exposure/case assessment)** | **RR (95% CI)** | **Matched/Adjusted factors** |
| --- | --- | --- | --- | --- |
| **Prospective studies** |  |  |  |  |
| Duell et al [6], 2013, Europe, NC-CS | 161/328,610  (35-70y), N/A | Nulliparous  1  2  3  ≥4  (Self-questionnaire/Cancer registry) | 1.00 (Ref)  1.01 (0.64-1.59)  1.03 (0.69-1.53)  0.92 (0.59-1.43)  1.15 (0.71-1.85) | Age, sex, study center, date and time of blood collection, fasting status, exogenous hormone use, smoking, education, alcohol consumption at baseline, red meat intake, BMI, and DM history |
| Lee et al [7], 2013, USA, CS | 323/118,164  (22-79y), 14 y | Nulliparous  1  2  3  ≥4  (Self-questionnaire/Cancer registry) | 1.00 (Ref)  1.13 (0.75-1.70)  1.10 (0.78-1.55)  0.98 (0.67-1.43)  0.94 (0.62-1.44) | Race/ethnicity, BMI, menopausal status, HT use, age at menarche, smoking status, and history of DM |
| Chang et al [9] ^†^, 2010, Taiwan, CS | 243/1,292,462 (N/A), 30 y | 1  2  ≥3  (Database/Cancer registry) | 1.00 (Ref)  0.69 (0.49-0.98)  0.64 (0.44-0.93) | Age at first childbirth, marital status, years of schooling, and birth place |
| Stevens et al [12], 2009, United Kingdom, CS | 1182/995,192 (mean 58.7/57.3y) 7.1 y | Nulliparous  1  2  3  ≥4  (Self-questionnaire/Cancer registry) | 1.00 (Ref)  0.88 (0.74-1.02)  0.78 (0.70-0.86)  0.81 (0.71-0.91)  0.84 (0.72-0.96) | Age, region, socioeconomic status, smoking, BMI, and DM |
|  |  |  |  | ***(Continued)*** |

**Table S1**

**(Continued)**

| **First author, publication year (reference), Country, Study design** | **Cases/subject (age), duration of follow up** | **Parity categories (exposure/case assessment)** | **RR (95% CI)** | **Matched/Adjusted factors** |
| --- | --- | --- | --- | --- |
| Heuch et al [13], 2008, Norway, CS | 449/63,090 (50-89y), 38 y | 1  2  3  4  ≥5  (Trained interviewer/Cancer registry) | 1.00 (Ref)  0.97 (0.69–1.35)  1.47 (1.03–2.09)  1.49 (0.98–2.26)  1.63 (1.01–2.64) | Age, birth cohort, county, and breastfeeding |
| Prizment et al [15], 2007, USA, CS | 228/37,459 (55-69y), 18 y | Nulliparous  1-2  3-4  ≥5  (Self-questionnaire/Cancer registry) | 1.00 (Ref)  1.14 (0.69-1.88)  1.10 (0.67-1.81)  1.09 (0.63-1.87) | Age |
| Lin et al [16] ^†^, 2006, Japan, CS | 154/63,273 (40-79y), 11y | 0-1  2-3  4-5  ≥6  (Self-questionnaire/Cancer registry) | 1.00 (Ref)  0.74 (0.38-1.43)  0.84 (0.40-1.75)  0.80 (0.31-2.11) | Age, pack-years of smoking, history of DM, history of gallstone/cholecystitis, and age at first birth |
| Navarro et al [18], 2005, Canada, CS | 187/89,835 (40-59y), 16.4y | Nulliparous  1-2  3-4  ≥5  (Self-questionnaire/Cancer registry) | 1.00 (Ref)  0.67 (0.43-1.06)  0.81 (0.53-1.24)  1.05 (0.64-1.74) | Age, cigarette smoking intensity, cigarette smoking duration, BMI, height, study center, and randomization group |
|  |  |  |  | ***(Continued)*** |

**Table S1**

**(Continued)**

| **First author, publication year (reference), Country, Study design** | **Cases/subject (age), duration of follow up** | **Parity categories (exposure/case assessment)** | **RR (95% CI)** | **Matched/Adjusted factors** |
| --- | --- | --- | --- | --- |
| Teras et al [17] ^†^, 2005, USA, CS | 1959/387,981 (mean N/A/60.5y), 18y | Nulliparous  1  2  3  4  5  6  ≥7  (Self-questionnaire/Cancer registry) | 1.00 (Ref)  0.99 (0.84-1.17)  0.96 (0.83-1.11)  1.10 (0.95-1.28)  0.96 (0.80-1.15)  0.78 (0.61-0.99)  0.88 (0.65-1.19)  0.76 (0.57-1.03) | Age, race, smoking history, education, personal history of DM, BMI, height, exercise, and family history of pancreatic cancer |
| Skinner et al [20], 2003, USA, CS | 243/115,474 (30-55y), 22y | Nulliparous  1-2  3-4  ≥5  (Self-questionnaire/Medical record) | 1.00 (Ref)  0.86 (0.55-1.36)  0.75 (0.48-1.17)  0.58 (0.34-0.98) | Age, time period, cigarette smoking, DM, BMI, and height |
| Karlson et al [23] ^‡^, 1998, Sweden, NC-CS | 1015/5073 (mean: 52.7/52.7y) | Nulliparous  1  2  3  4  ≥5  (Registry/Cancer registry) | 1.00 (Ref)  1.14 (0.89-1.45)  1.03 (0.81-1.31)  1.18 (0.89-1.57)  1.36 (0.96-1.94)  1.32 (0.85-2.05) | Age at diagnosis |
|  |  |  |  | ***(Continued)*** |

**Table S1**

**(Continued)**

| **First author, publication year (reference), Country, Study design** | **Cases/subject (age), duration of follow up** | **Parity categories (exposure/case assessment)** | **RR (95% CI)** | **Matched/Adjusted factors** |
| --- | --- | --- | --- | --- |
| **Case-control studies** |  |  |  |  |
| Lucenteforte et al [8], 2011, Italy, HC-CS | 285/713 (median 61/58y) | Nulliparous  1  2  3  ≥4  (Trained interviewer/Medical record) | 1.00 (Ref)  0.72 (0.45-1.16)  0.86 (0.55-1.33)  0.88 (0.53-1.46)  0.46 (0.26-0.85) | Study/center, age, education, area of residence, year of interview, history of DM, and tobacco smoking |
| Zhang et al [10], 2010, USA, HC-CS | 284/1096 (mean 57.5/57.0y) | Nulliparous  1-2  3-4  ≥4  (Trained interviewer/Medical record) | 1.00 (Ref)  1.5 (1.0-2.3)  1.4 (0.9-2.2)  1.5 (0.8-2.6) | Age, study center, race, interview year, years of education, BMI, cigarette, alcohol consumption, history of DM, age at menarche, age at first live birth, OC use, age at menopause, and hormone use |
| Duell et al [11] ^‡^, 2009, multicenter, PC-CS | 367/821 (28-87y) | Nulliparous  1  2  ≥3  (Trained interviewer/Medical record) | 1.00 (Ref)  1.35 (0.69-2.63)  1.38 (0.66-2.89)  1.26 (0.69-2.29) | Smoking, schooling, age, center, and type of interview with interaction terms between age and center and type of interview and center |
| Lo et al [14], 2007, Egypt, HC-CS | 194/194 (mean 56.4/54.4y) | 0-3  4-6  ≥7  (Trained interviewer/Medical record) | 1.00 (Ref)  1.3 (0.6-2.8)  0.5 (0.2-1.3) | Age, sex, residence, and active smoking |
|  |  |  |  | ***(Continued)*** |

**Table S1**

**(Continued)**

| **First author, publication year (reference), Country, Study design** | **Cases/subject (age), duration of follow up** | **Parity categories (exposure/ case assessment)** | **RR (95% CI)** | **Matched/Adjusted factors** |
| --- | --- | --- | --- | --- |
| Duell et al [19] ^‡^, 2005, USA, PC-CS | 241/818 (21-85y) | Nulliparous  1  2  3  ≥4  (Trained interviewer/Medical record) | 1.00 (Ref)  1.3 (0.75-2.2)  1.0 (0.63-1.6)  1.3 (0.81-2.1)  1.0 (0.63-1.7) | Sex, age, education, and smoking |
| Hanley et al [22], 2001, Canada, PC-CS | 112/1191 (N/A) | Nulliparous  1  2  3  ≥4  (Self-questionnaire/Cancer registry) | 1.00 (Ref)  0.61 (0.20-1.81)  0.94 (0.43-2.08)  0.89 (0.40-2.00)  0.51 (0.24-1.01) | Age, province, caloric intake, age at first menstruation, cigarette pack-years and maximum BMI |
| Kreiger et al [21], 2001, Canada, PC-CS | 52/233 (20-74y) | Nulliparous  1-2  ≥3  (Self-questionnaire/Cancer registry) | 1.00 (Ref)  0.69 (0.24-2.00)  0.22 (0.07-0.65) | Age, smoking status, BMI, tofu, dietary fat, age at menarche, age at menopause, OC use, estrogen replacement therapy, age at first full term pregnancy |
| Ji et al [24], 1996, China, PC-CS | 179/673 (median 65/61y) | 0-2  3-4  ≥5  (Trained interviewer/Cancer registry) | 1.00 (Ref)  1.33 (0.80-2.23)  1.88 (1.09-3.24) | Age, income, education, smoking, green tea drinking, respondent status and usual BMI |
|  |  |  |  | ***(Continued)*** |

**Table S1**

**(Continued)**

| **First author, publication year (reference), Country, Study design** | **Cases/subject (age), duration of follow up** | **Parity categories (exposure/ case assessment)** | **RR (95% CI)** | **Matched/Adjusted factors** |
| --- | --- | --- | --- | --- |
| Fernandez et al [25], 1995, Italy, HC-CS | 133/377 (median 62/59y) | Nulliparous  1  2  3  ≥4  (Trained interviewer/Medical record) | 1.00 (Ref)  0.6 (0.3-1.2)  0.8 (0.4-1.4)  0.8 (0.4-1.8)  0.5 (0.2-1.1) | Age, education, area of residence and smoking habit |
| Cantor et al [27], 1993, USA, PC-CS | 169/821 (N/A) | 1  2  3  4  ≥5  (Self-questionnaire/Cancer registry) | 1.00 (Ref)  0.7 (0.4-1.3)  1.1 (0.6-2.1)  0.6 (0.3-1.3)  0.5 (0.3-1.2) | Age, first degree relative of pancreatic cancer, cigarette smoking status, ever diagnosed with jaundice |
| Bueno et al [30], 1992, Netherlands, PC-CS | 82/252 (35-79y) | 1  2  3  ≥4  (Trained interviewer/Medical record) | 1.00 (Ref)  0.62 (0.23-1.69)  0.62 (0.22-1.74)  0.51 (0.21-1.25) | Age, response status and life-time smoking of cigarettes |
|  |  |  |  | ***(Continued)*** |

BMI: body mass index; CI: confidence interval; CS: cohort study; DM: diabetes mellitus; HC-CS: hospital-based case-control study; N/A: not available; NC-CS: nested case-control study; OC: oral contraceptive; OR: odds ratio; PC-CS: population-based case-control study; Ref: reference; RR: relative risk.

**†** Reported the risk estimate with pancreatic cancer mortality.

**‡** Recalculate the RR by the method proposed by Hamling et al [37].
